# Role of paan chewing and dietary habits in cervical carcinoma in Chennai, India

**DOI:** 10.1038/sj.bjc.6600902

**Published:** 2003-04-29

**Authors:** T Rajkumar, S Franceschi, S Vaccarella, V Gajalakshmi, A Sharmila, P J F Snijders, N Muñoz, C J LM Meijer, R Herrero

**Affiliations:** 1 Cancer Institute (WIA), 18 Sardar Patel Road, Adyar, Chennai 600 020, India; 2 International Agency for Research on Cancer, 150 cours Albert Thomas, F-69372 Lyon, cedex 08, France; 3 Epidemiological Research Center, Chennai 600 010, India; 4 Free University Medical Center, Postbus 7057, NL–1007 MB Amsterdam, the Netherlands; 5 Proyecto Epidemioloágico Guanacaste, Costa Rican Foundation for Health Sciences, San Joseá, Costa Rica

**Keywords:** paan chewing, vegetables, fruit, body mass index, cervical cancer

## Abstract

Non-viral factors contribute to human papillomavirus (HPV)-related cervical carcinogenesis. We investigated the role of paan chewing and dietary habits among 205 women with invasive cervical cancer (ICC) and 213 age-matched control women in Chennai, India. Odds ratios (OR) and 95% confidence intervals (CI) were computed by means of unconditional multiple regression, taking into account major correlates of ICC risk. Paan chewing showed a dose-dependent direct association with ICC (OR for ≥5 paan day^−1^=4.0; 95% CI 1.2–13.3). Among dietary habits, the highest *vs* lowest intake tertile for vegetables and fruit was associated with an OR of 0.5 (95% CI 0.2–1.0). Low education level and low body weight were also risk factors for ICC, but they did not account for the associations of paan chewing and low vegetable and fruit intake. In the analyses restricted to HPV-positive cases and controls, the inverse association with vegetable and fruit intake was confirmed. Conversely, the adverse influence of paan chewing on ICC risk seemed to be attributable to a higher prevalence of cervical HPV infection in women who chewed.

Infection with oncogenic types of human papillomavirus (HPV) has been established as the central cause of invasive cervical cancer (ICC) (Bosch *et al*, 2002). The probability of developing preinvasive and invasive cervical neoplasias is relatively low, given that between 10 and 50% of sexually active women have been found to carry cervical HPV infection (Bosch *et al*, 2002) and this probability depends upon other factors acting in conjunction with HPV. Cigarette smoking is associated with an approximately two-fold increase in the risk of ICC (Szarewski and Cuzick, 1998; Plummer *et al*, 2001), even in studies where stratification and adjustment for HPV infection and individual sexual habits excluded the possibility that smoking was a surrogate marker for such variables (Plummer *et al*, 2001). No information exists on whether smokeless tobacco (e.g. in some paan chewing) is also associated with ICC (IARC, 1985).

Several studies have shown that low vegetable and fruit intake (La Vecchia *et al*, 1988; Herrero *et al*, 1991) and low serum levels of various micronutrients (Potischman *et al*, 1991; Giuliano *et al*, 1997; Wideroff *et al*, 1998; Schiff *et al*, 2001; Sedjo *et al*, 2002) are associated with a moderate increase in the risk of invasive and preinvasive cervical tumours. Most studies on dietary habits and ICC were conducted, however, before the most sensitive, polymerase chain reaction (PCR)-based assays for HPV-DNA detection became available. They were, hence, not able to take the strong effect of HPV infection into account.

We, therefore, explored the role of paan chewing and dietary habits in ICC aetiology in Chennai (formerly Madras), Southern India, one of the areas with the highest ICC incidence rates (38.9/100 000; Parkin *et al*, 1997) worldwide, by means of a case–control study that could allow carefully for HPV infection.

## MATERIALS AND METHODS

This study is part of an international case–control study of ICC and HPV coordinated by the International Agency for Research on Cancer (IARC) (Muñoz *et al*, 2002). Between June 1998 and May 1999, 222 women with a diagnosis of ICC were interviewed at the Cancer Institute in Chennai, Southern India. On account of the high burden of ICC cases at the Cancer Institute, the first woman to be newly diagnosed with cervical cancer in each working day was asked to participate in the study and informed consent was obtained. Inclusion criteria were: (1) histological confirmation of ICC diagnosis, (2) no previous cancer treatment, and (3) lack of physical or mental impairments that would have made the interview impossible. In all, 17 women were excluded based on the revision of histological report: two had no neoplastic lesions, 14 women had cervical intraepithelial neoplasia (CIN) I or II, one had carcinoma *in situ*. Among 205 eligible ICC cases, the distribution by FIGO stage was the following: stage 1: 10.2%; stage 2: 44.9%; stage 3: 38.5%; stage 4: 6.3%. Squamous-cell carcinoma was diagnosed in 193 cases and adenocarcinoma/adenosquamous carcinoma in 12. In all, 12 cancer cases refused to provide cervical samples, although they were willing to be interviewed, leaving a total of 193 cervical cancer patients with cervical exfoliated cells available for HPV testing.

Control women were identified at the Cancer Institute among in-patients and visitors of patients other than women with cervical cancer, and were frequency matched by age with ICC cases within 5 years age groups. Exclusion criteria for control women included: (1) a diagnosis of anogenital tract cancers (i.e. cervix, vagina, vulva, anal canal), cancer of the breast, endometrium, ovary or colon, benign genital tumours and tobacco-related diseases (e.g. coronary heart disease, lung cancer or chronic bronchitis); (2) a history of hysterectomy or cervical conisation; and, as for ICC cases, (3) physical or mental problems. A total of 179 visitors and 37 in-patients were thus contacted. Of the eligible 216 controls, three declined to participate in the study and three refused to have a gynaecological examination. Thus, a sample of exfoliated cells from the cervix was available for 210 control women.

### Data and specimen collection

Each participant was administered a standardised questionnaire on socioeconomic status, sexual behaviour, reproductive history, contraceptive practices, smoking habits, genital hygiene, history of sexually transmitted infections and cervical cytological screening. Questions on weekly consumption of 21 foods or food groups, and summary questions on overall consumption of all vegetables and fruits were included. Information on height (cm) at time of interview and weight (kg) 2 years before interview was self-reported. Two trained female interviewers administered the questionnaire to all case and control women in the hospital.

After the interview, each consenting woman had a pelvic examination performed by a gynaecologist, who took two cervical scrapes with two Ayre spatulas and one endocervical brush. A Papanicolaou smear was prepared, and the remaining cells were eluted in phosphate-buffered saline (PBS), pelleted in PBS (2000 r.p.m. for 20 min at room temperature), and then stored at −70°C. A punch biopsy sample from the tumour (cases only) and a 10-ml sample of peripheral blood were also collected and stored at −70°C.

The study was approved by the ethical review committees of the IARC in Lyon and the Cancer Institute in Chennai.

### Detection of HPV DNA

To analyse the quality of target DNA, beta (*β*)-globin gene-specific primers were used. *β*-Globin-negative and HPV-negative samples (i.e. two ICC cases and 26 control women) were considered inadequate and excluded from HPV-related analyses, thus leaving 191 invasive cancer cases (179 squamous-cell carcinoma cases and 12 adeno/adenosquamous carcinoma cases) and 184 control women with adequate HPV-DNA results.

The presence of HPV DNA in cervical cells was assessed using general primer-mediated GP5+/6+ PCR. Polymerase chain-reaction positivity was assessed by means of hybridisation of PCR products in an enzyme immunoassay (EIA) using two HPV oligoprobe cocktails that together detect the following 36 HPV types: HPV 16, 18, 31, 33, 35, 39, 45, 51, 52, 56, 58, 59, 66, 68, 6, 11, 26, 34, 40, 42, 43, 44, 53, 54, 55, 57, 61, 70, 71 (equivalent to CP8061), 72, 73, 81 (equivalent to CP8304), 82 (IS39 and MM4 subtypes), 83 (equivalent to MM7), 84 (equivalent to MM8) and CP 6108. Probes and procedures used for EIA detection are described elsewhere (Jacobs *et al*, 2000). In addition, HPV-positivity was assessed by low-stringency Southern blot analysis of PCR products with a cocktail probe of HPV-specific DNA fragments. Subsequently, GP5+/6+ PCR was repeated on positive samples in triplicate to generate sufficient products for further typing. After pooling these PCR products, typing was performed using EIA and HPV type-specific oligoprobes for the HPV types described above (Jacobs *et al*, 1995).

Finally, E7 open-reading frame type-specific PCR assays for 14 high-risk HPV types (16, 18, 31, 33, 35, 39, 45, 51, 52, 56, 58, 59, 66, 68) (Walboomers *et al*, 1999) were applied to five ICC case specimens that were positive for *β*-globin but negative for HPV DNA by GP5+/GP6+ PCR. Reamplification of specimens with E7 was not done among control women, since integration of HPV DNA in the cellular genoma, and, therefore, loss or disruption of HPV L1 open-reading frame should not be found in women without cervical cancer (Walboomers *et al*, 1999).

### Statistical analyses

To estimate the risk of cervical cancer associated with various HPV types and the other risk factors, we calculated odds ratios (ORs) and 95% confidence intervals (CIs) by unconditional logistic regression. The ORs were adjusted for age and area of residence (Chennai and out of Chennai), education level, occupation, marital status, age at first marriage (which nearly always coincided with age at first sexual intercourse), number of pregnancies and husband's extramarital affairs, as indicated in the Tables. Only 4.9% of cases and 1.4% of control women reported more than one lifetime sexual partner and zero cases and 1.4% of control women reported oral contraceptive use. Therefore, these two variables were not included in the models. Approximate tertiles of weight, height, body mass index (BMI, weight kg/height, m^2^), and intake of foods or food groups were computed based on the combined distribution of cases and controls. Tests for linear trend were performed assigning an increasing score for each level of categorical variables. Scores were then fitted into the model as a continuous variable. In order to elucidate the influence of HPV infection, major analyses were repeated: (1) among all cases and controls, (2) among HPV-positive *vs* HPV-negative control women, and (3) among HPV-positive cases *vs* HPV-positive control women only.

## RESULTS

Table 1

**Table 1:** The distribution of 205 cases of cervical carcinoma and 213 controls with corresponding odds ratios (OR) and 95% confidence intervals (CI) by selected characteristics (Chennai, 1998–1999)

	**No. Cases**	**%**	**No. Controls**	**%**	**OR (95% CI)^b^**
Age (years)					
<45	70	34.1	73	34.3	—
45–49	44	21.5	44	20.6	—
⩾50	91	44.4	96	45.1	—

Human papilloma-virus DNA					
Negative	1	0.5	133	72.3	1^c^
Positive	190	99.5	51	27.7	387.7 (82.2–7443.8)

Education					
High	22	10.7	62	29.1	1^c^
Primary	22	10.7	48	22.5	1.42 (0.59–3.43)
None	161	78.5	103	48.4	2.69 (1.25–5.79)
*χ*^2^_1_ for trend					7.53; *P*=0.006

Occupation					
Housewife	34	16.6	99	46.5	1^c^
Farmer	140	68.3	74	34.7	4.33 (2.30–8.15)
Other	31	15.1	40	18.8	1.52 (0.70–3.32)

Marital status					
Married	143	69.8	201	94.4	1
Widow	55	26.8	12	5.6	8.11 (3.57–18.43)
Separated-divorced	7	3.4	0	0.0	

Age at first marriage/cohabiting (years)					
⩾21	20	9.8	36	16.9	1
19–20	39	19.0	41	19.3	1.23 (0.48–3.14)
17–18	53	25.9	69	32.4	0.76 (0.32–1.84)
15–16	45	22.0	32	15.0	0.74 (0.28–1.95)
<15	48	23.4	35	16.4	0.80 (0.31–2.10)
*χ*^2^_1_ for trend					0.92; *P*=0.337

Number of pregnancies					
<3	34	16.6	70	32.9	1
3–4	85	41.5	90	42.3	3.06 (1.54–6.10)
⩾5	86	42.0	53	24.9	4.99 (2.37–10.49)
*χ*^2^_1_ for trend					17.26; *P*<0.001
Husband's extramarital affairs^d^					
No	58	28.6	156	73.6	1
Uncertain/yes	145	71.4	56	26.4	6.99 (4.08–12.00)

a Some strata do not add up to the total because of missing values.

b Estimates from unconditional regression equations, including terms for age and area of residence, education, occupation, marital status, age at first marriage, number of pregnancies and husband's extramarital affairs.

c Reference category.

d Including prostitutes.

)shows the distribution of 205 cases of cervical carcinoma and 213 control women according to selected characteristics. Only one case was found to be HPV-DNA negative and the OR for HPV-DNA positivity was 388. The vast majority of HPV infections involved high-risk HPV types and no cases and 11 control women were infected only with low-risk types (data not shown). Cervical cancer cases reported a lower education level than control subjects (OR=2.7 for no education *vs* secondary school or more). Approximately half of the cases and controls combined reported to work in agriculture, and farmers showed an OR of 4.3 compared with housewives. Being a widow or divorced rather than currently married (OR=8.1), having had ⩾5 *vs* 1–2 pregnancies (OR=5.0), and reporting that one's husband had had (or probably had) extramarital affairs (OR=7.0) were associated with significantly increased risk of cervical cancer (Table 1).

In respect to paan chewing habits, 18.5% of cancer cases and 7.0% of control women reported to be ever chewers (OR=2.3; 95% CI: 1.0–5.3) (Table 2

**Table 2:** The distribution of 205 cases of cervical carcinoma and 213 controls with corresponding odds ratios (OR) and 95% confidence intervals (CI) by chewing and smoking habits, weight and height (Chennai, 1998–1999)

	**No. Cases**	**%**	**No. Controls**	**%**	**OR (95% CI)^b^**
Chewing habit					
Never	167	81.5	198	93.0	1^c^
Ever	38	18.5	15	7.0	2.30 (1.00–5.34)

Type of paan					
Without tobacco	10	4.9	6	2.8	2.64 (0.71–9.75)
With tobacco	28	13.6	9	4.2	2.13 (0.78–5.86)

No of paan/day					
<5	16	7.8	9	4.2	1.42 (0.49–4.14)
⩾5	22	10.7	6	2.8	4.00 (1.20–13.33)
*χ*^2^_1_ for trend					5.08; *P*=0.02

Smoking habit					
Never	203	99.0	213	100.0	1^c^
Ever	2	1.0	0	0.0	∞ (0.28–∞)

Weight (kg)					
⩾55	58	28.7	86	40.4	1^c^
50–54	79	39.1	75	35.2	1.56 (0.84–2.88)
⩽49	65	32.2	52	24.4	1.92 (0.98–3.75)
*χ*^2^_1_ for trend					3.72; *P*=0.06

Height (cm)					
⩾155	63	31.2	83	39.0	1^c^
151–154	59	29.2	66	31.0	1.14 (0.59–2.20)
⩽150	80	39.6	64	30.0	1.69 (0.89–3.23)
*χ*^2^_1_ for trend					2.56; *P*=0.11

a Some strata do not add up to the total because of missing values.

b Estimates from unconditional regression equations, including terms for age and area of residence, education, occupation, marital status, age at first marriage, number of pregnancies and husband's extramarital affairs.

c Reference category.

). The majority of chewers used paan with tobacco. No substantial difference in the ORs was found according to the presence of tobacco in paan. Women who reported to chew five or more paans per day showed a more elevated OR (4.0; 95% CI: 1.2–13.3) than women who reported less than 5 paans per day (1.4; 95% CI: 0.5–4.1) (*χ*^2^ for trend=5.08, *P*=0.02). Two cases and no controls reported to have ever smoked (Table 2).

The mean weight was 52 and 54 kg among cases and controls, respectively. Women who weighted less than 50 kg had an OR of 1.9 compared with women who weighted 55 kg or more (*χ*^2^_1_ for trend=3.72, *P*<0.06). A nonsignificant inverse association was found also for height (OR for <1.51 *vs* >1.54 m=1.7; *χ*^2^_1_ for trend=2.56, *P*=0.11).

Table 3

**Table 3:** Odds ratios (OR) and corresponding 95% confidence intervals (CI) for cervical carcinoma according to approximate intake tertile of selected foods or food groups; 205 cases and 213 controls (Chennai, 1998–1999)

	**Approximate intake tertile**			
		**OR (95% CI)^a^^,^^b^**		
**Food or food group**	**Servings (week)**	**II**	**III (highest)**	***χ*^2^_1_ for trend**

Fresh tomatoes	(<3: 3–4; ⩾5)	0.94 (0.33–2.71)	0.64 (0.25–1.66)	1.72; *P*=0.19
Green vegetables	(0; <2; ⩾2)	1.68 (0.75–3.73)	0.59 (0.22–1.59)	0.08; *P*=0.78
Cruciferae	(<3; 3–4; ⩾5)	0.75 (0.32–1.72)	0.73 (0.36–1.49)	0.63; *P*=0.43
Carrots	(<3; 3–4; ⩾5)	0.66 (0.28–1.58)	0.64 (0.31–1.35)	1.08; *P*=0.30
Pulses	(<5; 5; ⩾6)	0.74 (0.39–1.40)	0.38 (0.17–0.82)	5.86; *P*=0.02
Fruit juices	(0; ⩾1)	0.61 (0.18–2.11)	—	
Apples or pears	(0; ⩾1)	0.90 (0.28–2.91)	—	
Citrus fruit	(<1; ⩾1)	0.59 (0.25–1.40)	—	
Bananas	(<1; 1; ⩾2)	0.77 (0.38–1.54)	0.30 (0.11–0.78)	5.63; *P*=0.02
Milk	(<1; 1; ⩾2)	0.98 (0.52–1.83)	0.67 (0.33–1.36)	1.21; *P*=0.27
Yoghurt	(<1; 1–5; ⩾7)	1.12 (0.61–2.06)	0.63 (0.30–1.31)	1.28; *P*=0.26
Bread	(0; 0.5; ⩾1)	1.68 (0.93–3.01)	2.04 (0.82–5.04)	4.15; *P*=0.04
Rice and pasta	(<7; ⩾7)	1.93 (0.57–6.47)	—	
Maize	(0; >0)	0.13 (0.01–1.64)	—	
Eggs	(0; <2; ⩾2)	1.30 (0.52–3.24)	0.63 (0.19–2.04)	0.80; *P*=0.37
Fish	(0; <1 ⩾1)	1.24 (0.44–3.46)	1.28 (0.60–2.71)	0.36; *P*=0.55
Ham and salami	(0; <1; ⩾1)	1.94 (0.65–5.81)	1.47 (0.63–3.42)	1.28; *P*=0.26
Meat	(0; <1; ⩾1)	1.14 (0.39–3.36)	1.41 (0.59–3.37)	0.76; *P*=0.38
Cakes and desserts	(0; ⩾1)	1.94 (0.42–8.89)	—	
Fat for seasoning	(seed *vs* palm/coconut oil)	0.81 (0.30–2.17)	—	
Fat for cooking	(seed *vs* palm/coconut oil)	0.92 (0.36–2.37)	—	

a Reference category is at the lowest intake tertile.

b Estimates from unconditional regression equations, including terms for age, area of residence, education, occupation, marital status, age at first marriage, number of pregnancies and husband's extramarital affairs.

shows ORs by intake tertile of selected foods or food groups and cervical cancer. Significant inverse associations emerged for pulses (OR in the highest *vs* lowest intake tertile=0.4)and bananas (OR=0.3). The inverse associations with intake of fresh tomatoes, carrots, cruciferae and other citrus fruit were not statistically significant. There was no indication that the consumption of green vegetables, apples or pears, rice, milk, yogurt, egg, fish, meat, cakes or desserts and various oils affected ICC risk. A direct association was found for the intake of bread (OR=2.0).

Table 4

**Table 4:** Odds ratios (OR) and corresponding 95% confidence intervals (CI) according to selected characteristics among all cases and controls; controls only, by HPV DNA; and HPV DNA-positive cases and controls only (Chennai, 1998–1999)

	**All**		**Controls**		**HPV+**	
	**Cases *vs* controls**		**HPV+ *vs* HPV−**		**Cases *vs* controls**	
	**Ca/Co**	**OR (95% CI)**	**HPV+/**−	**OR (95% CI)**	**Ca/Co**	**OR (95% CI)**
Chewing habit						
Never	167/198	1	45/126	1	154/4	1
					5	
Ever	38/15	2.36 (0.99–5.60)	6/7	2.12 (0.56–8.02)	36/6	1.06 (0.27–4.10)
						
BMI						
⩾23.3^a^	51/86	1	14/59	1	50/14	1
21.5–23.3	75/63	1.66 (0.85–3.25)	19/35	2.21 (0.86–5.67)	71/19	1.04 (0.30–3.53)
<21.5	76/64	1.63 (0.83–3.21)	18/39	2.03 (0.81–5.04)	66/18	1.04 (0.32–3.42)
*χ*^2^_1_ for trend	1.88; *P*=0.17	2.28; *P*=0.13	0.00; *P*=0.95			
						
Vegetable and fruit intake (servings week^−1^)						
<6	76/42	1	10/23	1	67/10	1
6	77/83	0.68 (0.34–1.33)	19/55	0.83 (0.28–2.40)	74/19	0.88 (0.26–3.03)
⩾7	52/88	0.48 (0.24–0.98)	22/55	0.86 (0.30–2.43)	49/22	0.37 (0.11–1.22)
*χ*^2^_1_ for trend	4.10; *P*=0.04	0.05; *P*=0.82	3.05; *P*=0.08			

a Estimates from unconditional regression equations, including terms for age, area of residence, occupation, marital status, age at first marriage, number of pregnancies, husband's extramarital affairs and all listed variables. HPV=human papillomavirus. Ca=cases. Co=controls

shows the associations between ICC and paan chewing, BMI and overall vegetable and fruit intake according to the fully adjusted model in three different types of comparisons (see Statistical Analyses). Among all study women, paan chewing (ever *vs* never OR=2.4) and vegetable and fruit intake (summary question) (highest *vs* lowest intake tertile OR=0.5) were associated with ICC risk. In the analyses restricted to HPV-positive women, however, only the association with low vegetable and fruit intake (OR=0.4) was confirmed, even if it became of borderline statistical significance. Conversely, paan chewing was not related to ICC risk among HPV-positive women but showed a hint of direct association with HPV infection among control women.

## DISCUSSION

This is the first report of an association between paan chewing and cervical cancer. The topic is of interest in the light of the well-established association between ICC and tobacco smoking (Szarewsky and Cuzick, 1998). The mechanisms involved in the tobacco-cervical cancer link are not well understood, but some of them (e.g. high concentration of nicotine, cotinine and tobacco-specific nitrosamines in cervical mucus, impairment of immune function, accumulation of free radicals, etc, Szarewsky and Cuzick, 1998) may not require tobacco burning. Aqueous extracts of betel quids and arecanuts that are generally used to make paan produced carcinomas of the cheek pouch and fore-stomach of rodents after subcutaneous or intragastric administration (IARC, 1985). Chewing is common among women in South-East Asia and the South Pacific Islands (IARC, 1985) where cervical cancer rates are very high (Parkin *et al*, 1997), and, in Southern India, paan chewing has been found to account for 87% of oral cancer in women (Balaram *et al*, 2002).

In addition to paan chewing, low education, occupation in farming, low weight and low intake of vegetables and fruit were risk factors for cervical cancer in our study. An excess of cervical cancer among low socioeconomic class women is one of the most consistent findings of epidemiological studies on this tumour (Schiffman *et al*, 1996). Although the socioeconomic gradient in cervical cancer is now chiefly attributed to a lack of adequate screening, it has been present long before the spread of Papanicolaou smears in developed countries and must have explanations other than screening in our study since only one woman (a case) in Chennai reported to have ever had a Papanicolaou smear.

A possible correlate of low social class is poor nutritional status. The inverse relation we found between ICC risk and vegetable and fruit intake, after allowing for education level and occupation, may support this possibility. Although we tried to obtain a woman's weight 2 years before cancer diagnosis or interview (controls), the apparent adverse effect of weight and BMI must be interpreted with caution, as it may be a consequence rather than a risk factor for cervical cancer. The findings on height, although not statistically significant, point to a possible influence of nutritional deficiencies in childhood and adolescence that might have prevented some women from attaining their full height. Unfortunately, the reliability of self-reported weight and height in India is unknown.

Our present evaluation of dietary habits also has limitations, since we have been using a short frequency questionnaire that included 21 indicator foods or food groups only and we did not explore serving size. Furthermore, the questionnaire had not been validated in India, and it was especially difficult to capture any association with staple foods like rice, which are eaten daily by the majority of the population, and with food groups which are eaten rarely (e.g, apples or pears and citrus fruit) or as ingredients of complex recipes (e.g, meat, green vegetables). No reliable translation into specific macro- and micronutrients has been possible, but in the summary questions on overall intake of all vegetables and fruit, ICC cases did report a significantly lower intake than control women, in agreement with findings of some previous reports (La Vecchia *et al*, 1988; Herrero *et al*, 1991).

The greatest challenge in the interpretation of our findings is, however, to rule out the possibility that the observed associations with paan chewing and low vegetable and fruit intake are not mere correlates of sexual habits and, most important, HPV infection. These associations were somewhat weakened, but were still statistically significant after adjustment for their possible mutual confounding effect and for all the major correlates of ICC risk in our study. HPV infection was found in all but one case and 28% of controls, and it involved, in the vast majority of women, high-risk HPV types.

The separate analysis of risk factors for HPV infection (among control women only) and for cervical cancer among HPV-positive cases and HPV-positive controls can help to elucidate whether these influence chiefly the acquisition and persistence of HPV infection or the progression from HPV infection into malignant cervical lesions (Muñoz *et al*, 2002). The comparison of ORs in Table 4 suggests that low intake of vegetables and fruit is unrelated to HPV infection, but is directly associated with ICC risk among HPV-positive women. These findings are in agreement with those of Wideroff *et al* (1998) and Sedjo *et al* (2002), who reported no association between intake of various vitamins and HPV infection. Paan chewing showed some, although nonsignificant, association with the prevalence of HPV infection among controls, but no association with ICC risk among HPV-positive women. A case–control study from the United Kingdom (Deacon *et al*, 2000) showed that tobacco smoking was associated with cervical intraepithelial neoplasia (CIN) III among HPV-positive women, but not with HPV-positivity among control women.

Unfortunately, the subgroup analyses in our study are based on small numbers of women and have very broad confidence intervals. However, the analyses restricted to HPV-positive women support the possibility that low intake of vegetables and fruit contributes to the progression from HPV infection to cervical cancer. With respect to paan chewing, either its adverse effect on cervical cancer is real and, possibly, mediated by a decreased ability of women who chew to clear HPV infection, or the habit of chewing is a surrogate marker for increased HPV exposure. Future prospective studies could evaluate the influence of paan chewing in the natural history of HPV infection and the development of preinvasive cervical lesions.

## References

[bib1] Balaram P, Sridhar H, Rajkumar T, Vaccarella S, Herrero R, Nandakumar A, Ravichandran K, Ramdas K, Sankaranarayanan R, Gajalakshmi V, Muñoz N, Franceschi S (2002) Oral cancer in Southern India: the influence of smoking, drinking, paan-chewing and oral hygiene. Int J Cancer 98: 440–445 11920597 10.1002/ijc.10200

[bib2] Bosch FX, Lorincz A, Muñoz N, Meijer CJLM, Shah KV (2002) The causal relation between human papillomavirus and cervical cancer. J Clin Pathol 55: 244–265 11919208 10.1136/jcp.55.4.244 PMC1769629

[bib3] Deacon JM, Evans CD, Yule R, Desai M, Binns W, Taylor C, Peto J (2000) Sexual behaviour and smoking as determinants of cervical HPV infection and of CIN3 among those infected: a case–control study nested within the Manchester cohort. Br J Cancer 88: 1565–1572 10.1054/bjoc.2000.1523 PMC2363425 11076670

[bib4] Giuliano AR, Papenfuss M, Nour M, Canfield LM, Schneider A, Hatch K (1997) Antioxidant nutrients: associations with persistent human papillomavirus infection. Cancer Epidemiol Biomark Prev 6: 917–923 9367065

[bib5] Herrero R, Potishman N, Brinton LA, Reeves WC, Brenes MM, Tenorio F, de Britton RC, Gaitan E (1991) A case–control study of nutrient status and invasive cervical cancer. I. Dietary indicators. Am J Epidemiol 134: 1335–1346 1755447 10.1093/oxfordjournals.aje.a116036

[bib6] IARC (1985) Tobacco habits other than smoking: betel-quid and areca-nut chewing; and some related nitrosamines. Monographs on the Evaluation of the Carcinogenic Risks to Humans, Vol. 37. Lyon: International Agency for Research on Cancer. 3866741

[bib7] Jacobs MV, de Roda Husman AM, van den Brule AJC, Snijders PJF, Meijer CJLM, Walboomers JMM (1995) Group-specific differentiation between high- and low-risk human papillomavirus genotypes by general primer-mediated PCR and two cocktails of oligonucleotide probes. J Clin Microbiol 33: 901–905 7790457 10.1128/jcm.33.4.901-905.1995 PMC228064

[bib8] Jacobs MV, Walboomers JM, Snijders PJ, Voorhorst FJ, Verheijen RH, Fransen-Daalmeijer N, Meijer CJ (2000) Distribution of 37 mucosotropic HPV types in women with cytologically normal cervical smears: the age-related patterns for high-risk and low-risk types. Int J Cancer 87: 221–227 10861478

[bib9] La Vecchia C, Decarli A, Fasoli M, Parazzini F, Franceschi S, Gentile A, Negri E (1988) Dietary vitamin A and the risk of intraepithelial and invasive cervical neoplasia. Gynecol Oncol 30: 187–195 3371743 10.1016/0090-8258(88)90023-6

[bib10] Muñoz N, Franceschi S, Bosetti C, Moreno V, Herrero R, Smith J, Shah KV, Meijer CJLM, Bosch FX for the IARC Multicentric Cervical Cancer Study Group (2002) Role of parity and human papillomavirus in cervical cancer: the IARC multicentric case–control study. Lancet 359: 1093–1101 11943256 10.1016/S0140-6736(02)08151-5

[bib11] Parkin DM, Whelan SL, Ferlay J, Raymond L, Young J (1997) Cancer Incidence in Five Continents, Vol. VII. Lyon: International Agency for Research on Cancer.

[bib12] Potischman N, Herrero R, Brinton LA, Reeves WC, Stacewicz-Sapuntzakis M., Jones CJ, Brenes MM, Tenorio F, de Britton RC, Gaitan E (1991) A case–control study of nutrient status and invasive cervical cancer. II. Serologic indicators. Am J Epidemiol 134: 1347–1355 1755448 10.1093/oxfordjournals.aje.a116037

[bib13] Plummer M, Herrero R, Bosch FX, Meijer CJLM, Franceschi S, Muñoz N, IARC Multicenter Cervical Cancer Study Group (2001) Smoking and Cervical Cancer: Pooled Analysis of a Multicentric Case-control Study. Proceedings, 19th International Papillomavirus Conference, Florianopolis, O-193. p. 214

[bib14] Schiff MA, Patterson RE, Baumgartner RN, Masum M, van Asselt-King L, Wheeler CM, Becker TM (2001) Serum carotenoids and risk of cervical intraepithelial neoplasia in Southwestern American Indian women. Cancer Epidemiol Biomark Prev 10: 1219–1222 11700272

[bib15] Schiffman MH, Brinton LA, Devesa SS, Fraumeni Jr JF (1996) Cervical cancer. In: Cancer Epidemiology and Prevention, Second Edition, Schottenfeld D, Fraumeni Jr JF (eds) pp. 1090–1116. Oxford University Press: Oxford.

[bib16] Sedjo RL, Inserrra P, Abrahamsen M, Harris RB, Roe DJ, Baldwin S, Giuliano AR (2002) Human papillomavirus persistence and nutrients involved in the methylation pathway among a cohort of young women. Cancer Epidemiol Biomark Prev 11: 353–359 11927495

[bib17] Szarewski A, Cuzik J (1998) Smoking and cervical neoplasia: a review of the evidence. J. Epidemiol Biostat 3: 229–256

[bib18] Walboomers JMM, Jacobs MV, Manos MM, Bosch FX, Kummer JA, Shah KV, Snijders PJF, Peto J, Meijer CJLM, Muñoz N (1999) Human papillomavirus is a necessary cause of invasive cervical cancer worldwide. J Pathol 189: 12–19 10451482 10.1002/(SICI)1096-9896(199909)189:1<12::AID-PATH431>3.0.CO;2-F

[bib19] Wideroff L, Potischman N, Glass AG, Greer CE, Manos MM, Scott DR, Burk RD, Sherman ME, Wacholder S, Schiffman M (1998) A nested case–control study of dietary factors and the risk of incident cytological abnormalities of the cervix. Nutr Cancer 30: 130–136 9589431 10.1080/01635589809514652

